# Supplementary material for: Patient-Reported Outcomes of Palbociclib Plus Exemestane with GnRH Agonist versus Capecitabine in Premenopausal Women with Hormone Receptor-Positive Metastatic Breast Cancer: A Prospective, Open-Label, Randomized Phase ll Trial (KCSG-BR 15-10)
Source: Cancers (Basel). 2020 Nov 5;12(11):3265. doi: 10.3390/cancers12113265 (PMC7694364; doi:10.3390/cancers12113265)
Supplement: Supplementary file 1 [file cancers-12-03265-s001.pdf]

Supplementary Materials:

Patient-Reported Outcomes of Palbociclib Plus Exemestane with GnRH Agonist versus Capecitabine in Premenopausal Women with Hormone Receptor-Positive Metastatic Breast Cancer: A Prospective, Open-Label, Randomized Phase ll Trial (KCSG-BR 15-10)

Soohyeon Lee, Seock-Ah Im, Gun Min Kim, Kyung Hae Jung, Seok Yun Kang, In Hae Park, Jee Hyun Kim, Kyoung Eun Lee, Hee Kyung Ahn, Moon Hee Lee, Hee-Jun Kim, Han Jo Kim, Jong In Lee, Su-Jin Koh, Yeon Hee Park and Korean Cancer Study Group (KCSG)

Table S1. Baseline characteristics between palbociclib plus exemestane with GnRH agonist and capecitabine arm.

	**Palbociclib plus Endocrine therapy (*n* = 92)**	**Capecitabine (*n* = 86)**
Age in years; median (range)	44 (31–58)	44 (28–53)
BMI; median (range)	22 (15–30)	22 (14–35)
Hormone receptor status		
ER+/PR+	70 (76.1%)	64 (74.4%)
ER+/PR−	22 (23.9%)	22 (25.6%)
ECOG performance status		
0	54 (58.7%)	48 (55.8%)
1–2	38 (41.3%)	38 (44.2%)
Metastatic sites		
Bone only	22 (23.9%)	18 (20.9%)
Visceral organs	45 (48.9%)	43 (50.0%)
lung	29 (31.5%)	22 (25.6%)
Liver	17 (18.5%)	19 (22.1%)
DFS from the 1st diagnosis of stage I–III BC (*N* = 124)	*N* = 64	*N* = 60
<24 months	12 (13.0%)	15 (17.4%)
≥24 months	52 (56.5%)	45 (52.3%)
Initially stage IV	28 (30.4%)	26 (30.2%)
Tamoxifen resistance (including recurrence within 1 year after completion of adjuvant tamoxifen)		
Yes	76 (82.6%)	77 (89.5%)
No	16 (17.4%)	9 (10.5%)
Prior cytotoxic chemotherapy for MBC		
Yes	22 (23.9%)	18 (20.9%)
No	70 (76.1%)	68 (79.1%)
Previous lines of treatment for MBC		
0	46 (50.0%)	45(51.2%)
1	30 (32.6%)	30(52.3%)
2	16 (17.4%)	11 (12.8%)

Prior (neo)adjuvant treatment (N = 124)	N = 64	N = 60
Cytotoxic chemotherapy	58 (90.1%)	50 (83.3%)
Anthracycline based	16 (25.0%)	11 (18.3%)
Anthracycline and taxane based	40 (62.5%)	36 (60.0%)
Others	2 (3.1%)	3 (5.0%)
Endocrine treatment	60 (93.8%)	55 (91.7%)
Tamoxifen	43 (67.2%)	47 (78.3%)
Tamoxifen + GnRH agonist	17 (26.6%)	8 (13.3%)

BMI = body mass index; ER = estrogen receptor; PR = progesterone receptor; DFS = disease free survival; BC = breast cancer; MBC = metastatic breast cancer.

Table S2. Comparison between two treatment arms of change pattern according to time of each subscale scores by analysis using generalized estimating equations.

	**Parameter**	**Level1**	**Estimate**	**Standard Error**	***p* value**
Global health status	ARMCD	ArmA vs ArmB	5.3442	2.9061	0.0659
	week_i		0.0064	0.0565	0.9100
	week_i × RMCD	ArmA vs ArmB	−0.0333	0.0678	0.6230
Physical functioning	ARMCD	ArmA vs ArmB	3.6421	2.7033	0.1779
	week_i		0.0225	0.0474	0.6353
	week_i × ARMCD	ArmA vs ArmB	0.0095	0.0549	0.8619
Role functioning	ARMCD	ArmA vs ArmB	5.8515	3.5271	0.0971
	week_i		−0.0189	0.0598	0.7524
	week_i × ARMCD	ArmA vs ArmB	0.0028	0.0696	0.9675
Emotional functioning	ARMCD	ArmA vs ArmB	1.3662	2.6889	0.6114
	week_i		−0.0707	0.073	0.3332
	week_i × ARMCD	ArmA vs ArmB	0.0775	0.0813	0.3405
Cognitive functioning	ARMCD	ArmA vs ArmB	2.593	2.4358	0.2871
	week_i		0.0234	0.0501	0.6412
	week_i × ARMCD	ArmA vs ArmB	−0.0769	0.0601	0.2010
Social functioning	ARMCD	ArmA vs ArmB	7.199	3.3151	0.0299
	week_i		−0.0246	0.0743	0.7409
	week_i × ARMCD	ArmA vs ArmB	0.0068	0.0901	0.9396
Fatigue	ARMCD	ArmA vs ArmB	−3.2795	3.0029	0.2748
	week_i		0.0595	0.0633	0.3471
	week_i × ARMCD	ArmA vs ArmB	−0.0569	0.0709	0.4222
Nausea and vomiting	ARMCD	ArmA vs ArmB	−7.081	2.1759	0.0011
	week_i		−0.0745	0.0405	0.0660
	week_i × ARMCD	ArmA vs ArmB	0.0695	0.0482	0.1493
Pain	ARMCD	ArmA vs ArmB	−6.8937	3.3473	0.0394
	week_i		−0.1107	0.0525	0.0349
	week_i × ARMCD	ArmA vs ArmB	0.0804	0.0658	0.2215
Dyspnea	ARMCD	ArmA vs ArmB	−0.8068	2.9748	0.7862
	week_i		−0.0292	0.0498	0.5573
	week_i × ARMCD	ArmA vs ArmB	−0.0126	0.0637	0.8430
Insomnia	ARMCD	ArmA vs ArmB	4.102	3.8952	0.2923
	week_i		0.0937	0.072	0.1933
	week_i × ARMCD	ArmA vs ArmB	−0.0626	0.0866	0.4702

Appetite loss	ARMCD	ArmA vs ArmB	−4.7466	3.0746	0.1226
	week_i		−0.0667	0.0544	0.2204
	week_i × ARMCD	ArmA vs ArmB	−0.0155	0.0647	0.8112
Constipation	ARMCD	ArmA vs ArmB	−1.3192	2.9857	0.6586
	week_i		−0.0357	0.0546	0.5131
	week_i × ARMCD	ArmA vs ArmB	0.0438	0.0692	0.5261
Diarrhea	ARMCD	ArmA vs ArmB	−8.3505	2.1837	0.0001
	week_i		0.1238	0.0547	0.0237
	week_i × ARMCD	ArmA vs ArmB	−0.1153	0.0635	0.0696
Total Score	ARMCD	ArmA vs ArmB	3.7713	2.1344	0.0772
	week_i		−0.0022	0.039	0.9553
	week_i × ARMCD	ArmA vs ArmB	0.0068	0.0455	0.8806

ARMCD = planned arm code.
